# Development and Evaluation of a Community Surveillance Method for Estimating Deaths Due to Injuries in Rural Nepal

**DOI:** 10.3390/ijerph18178912

**Published:** 2021-08-24

**Authors:** Santosh Bhatta, Julie Mytton, Elisha Joshi, Sumiksha Bhatta, Dhruba Adhikari, Sunil Raja Manandhar, Sunil Kumar Joshi

**Affiliations:** 1 Faculty of Health and Applied Sciences, School of Health and Social Wellbeing, University of the West of England, Bristol BS16 1QY, UK; julie.mytton@uwe.ac.uk; 2 Nepal Injury Research Centre, Department of Community Medicine, Kathmandu Medical College, Kathmandu P.O. Box 21266, Nepal; ejoshi03@gmail.com (E.J.); bhattasumiksha10@gmail.com (S.B.); drsunilkumarjoshi@gmail.com (S.K.J.); 3 Mother and Infant Research Activities (MIRA), Kathmandu P.O. Box 921, Nepal; dhrubaadhikari@mira.org.np (D.A.); s.manandhar@mira.org.np (S.R.M.)

**Keywords:** injury death, community surveillance, program evaluation, developing countries

## Abstract

Almost 10% of global deaths are secondary to injuries, yet in the absence of routine injury surveillance and with few studies of injury mortality, the number and cause of injury deaths in many countries are not well understood. This study aimed to develop and evaluate the feasibility of a method to identify injury deaths in rural Nepal. Working with local government authorities, health post staff and female community health volunteers (FCHVs), we developed a two-stage community fatal injury surveillance approach. In stage one, all deaths from any cause were identified. In stage two, an interview with a relative or friend gathered information about the deceased and the injury event. The feasibility of the method was evaluated prospectively between February 2019 and January 2020 in two rural communities in Makwanpur district. The data collection tools were developed and evaluated with 108 FCHVs, 23 health post staff and two data collectors. Of 457 deaths notified over one year, 67 (14.7%) fatal injury events were identified, and interviews completed. Our method suggests that it is feasible to collect data on trauma-related deaths from rural areas in Nepal. These data may allow the development of injury prevention interventions and policy.

## 1. Introduction

Injuries claim over five million lives annually, accounting for nine percent of the world’s total deaths [1]. More than 90% of deaths secondary to injury occur in low-and middle-income countries (LMICs), where preventive efforts are limited, and healthcare systems are least prepared to meet the challenge [1]. According to the Global Burden of Disease (GBD) study, in 2017, the death rates due to injury in Nepal (56.3/100,000) were estimated to be almost double that of high-income countries such as the United Kingdom (31.5/100,000) [2]. Of an estimated 182,751 deaths from all causes in Nepal, 9.2% (16,831) were due to injuries. While the overall mortality rate from injuries has decreased over the last two decades, the proportion of deaths due to injuries has increased by 2.9% during the same period [2].

Nepal is classified by the United Nations as one of the ‘least developed’ countries of the world [3], where about 80% of the country’s population live in rural areas [4]. The burden of injuries is unequally distributed and mostly affects lower socioeconomic groups [5]. Those living in rural areas are more likely to be disadvantaged, having lower levels of education, employment and income from work. Additional to their higher risk of being injured [6], they also have reduced access to healthcare and a consequent higher risk of suffering worse outcomes when an injury occurs. The injuries can place an economic burden on families and widen socioeconomic inequalities across society [7]. Injury surveillance can support country-level understanding of the burden of injuries, injury risks and population groups at risk for specific types of injury. Thus, surveillance can help inform the development of injury prevention interventions, which might facilitate changes in health systems, policy and practice [8].

In many countries, civil registration systems provide data on cause of death. Death registration is compulsory in Nepal, but there is no obligation to register the death within a specified period, and the cause of death is reported by the next of kin, not a health professional. Due to the lack of effective implementation of death registration, the available data is of limited use [9]. Nepal also lacks robust non-fatal injury data. The annual report of the Department of Health Services (DoHS) is the primary source of national-level data estimating the burden of diseases in Nepal [4]. These reports are based on the data collected through the Health Management Information System (HMIS) from health institutions across the country. Historically, published reports have focused on communicable diseases, maternal and nutritional data, and injuries have never been the focus of a DoHS report. There have been few nationally representative surveys to understand the mortality pattern of major forms of injuries in Nepal, the most recent being in 2006 using data from the 2001 census [10]. Most publications reporting injury outcomes are hospital case series and a few cross-sectional studies. These studies are at high risk of ascertainment bias and underreport the actual burden of injury [11]. In the absence of effective death registration or routine fatal injury monitoring systems, the number and cause of injury deaths in rural Nepal are not well understood.

In rural areas of Nepal, health services are delivered primarily through the local health facilities, such as health posts or primary healthcare centres, and with the involvement of female community health volunteers (FCHVs). FCHVs are part of many community-based health programmes and the government healthcare system [12]. Working with a local Health Coordinator, they act as a bridge between families, communities, health facilities and local government. They report demographic health data at monthly meetings in the local health facility [4]. Their practice of collecting community health data and meeting monthly with other FCHVs across the ward at the local health post means they are well positioned to identify and collate information on community deaths.

A small number of studies have attempted to estimate community deaths in Nepal, and these have used verbal autopsy methods [13] in suburban areas in order to identify the causes of neonatal deaths [14] or stillbirths [15]. Unlike these studies, we aimed to develop and evaluate a method for identifying, assessing and monitoring trends in injury deaths that has the potential to be embedded alongside existing routine health information systems in Nepal.

Therefore, this study aimed to develop and evaluate the feasibility of a method to identify and record injury deaths in rural areas of Nepal, and identify potential future actions.

## 2. Materials and Methods

### 2.1. Development of a Surveillance Method

#### 2.1.1. Two-Stage Data Collection Approach

**Figure 1:**
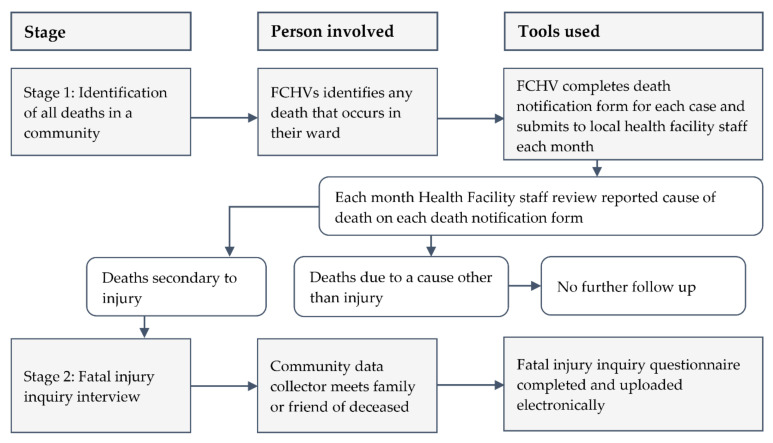
Overview of the two-stage data collection approach. (FCHV = Female Community Health Volunteers).

Working with local government authorities, health facility staff and FCHVs, we developed and refined a two-stage method of community fatal injury surveillance, as described in Figure 1.

Stage 1: Identification of all deaths—Local FCHVs were trained to identify and record all deaths occurring in the communities where they normally work. To respect traditional mourning periods, bereaved families were not visited by the FCHV until 15–30 days after the death, depending on mourning practices for that community. After taking consent from the next of kin, the FCHVs recorded demographic details about the person who had died, and a free text statement about what had happened to the person when they died, on a death notification form. The FCHV submitted the form to the staff of their local health facility during their monthly meetings. The focal health facility staff checked that the forms had been correctly completed, categorised the cause of the death as injury/not injury based on the information collected by FCHV and stored the forms securely. FCHVs were given 400 Nepalese rupees (approximately US $3) for each death notification form submitted.

Stage 2: Fatal injury inquiry interview—Trained data collectors visited the health facility every month to collect death notification forms from the health facility staff. The data collectors verified the death as a result of injury or other cause, using the guidance provided to them by the research team. Any uncertainty regarding whether a death should be identified as an injury death was resolved through discussion with the research office. The data collectors then approached the family of the deceased to negotiate a convenient time and location for an interview. A family member who was with the deceased at the time of the injury that resulted in the death, or someone who witnessed the death of the injured person, was interviewed. The interviewees were encouraged to involve other members of the family and friends where this was felt to be helpful. Interviews were conducted within three months of the injury event to maximise the completeness and quality of the information collected and reduce the potential for recall bias [16].

#### 2.1.2. Data Collection Tools

Development of the death notification form and fatal injury questionnaire was informed by data collection tools published by the World Health Organisation (WHO, Geneva, Switzerland) for verbal autopsies [13] and drew on the experience of conducting two community-based household injury surveys in Nepal between 2011 and 2016 [17,18]. We consulted researchers at the Centre for Injury Prevention and Research Bangladesh (CIPRB, Dhaka, Bangladesh) to learn from their experience of conducting a verbal autopsy study of maternal deaths [19]. The death notification form was paper-based, and captured information about the deceased and the contact details of the FCHV recording the death.

The fatal injury inquiry questionnaire was designed to capture anonymous detailed information on the person who died, and the circumstances of the injury event leading to death. Both direct and underlying causes of death were captured. For example, if a person died from rabies following a dog bite, rabies was recorded as the direct cause of death, and the dog bite as the underlying cause of death. The data collector recorded information from the informant directly onto a tablet computer that had been pre-loaded with the fatal injury inquiry questionnaire using Research Electronic Data Capture (REDCap, Vanderbilt University, Nashville, TN, USA) software [20]. The death notification form and fatal injury questionnaire are available as Supplementary Material (Tables S1 and S2).

#### 2.1.3. Data Management

Death notification forms were allocated a unique identifying number and de-identified data were entered into a Microsoft Access 2019 database (Microsoft Corporation) on a password-protected computer at the field office. Anonymous data, linked to the death notification form using the unique ID, and captured electronically during the fatal injury inquiry interview, were automatically uploaded to a REDCap database at the University of Bristol, UK (https://www.project-redcap.org/). Access to the data was restricted to members of the study team who checked for data completeness and resolved any queries with the data collector.

### 2.2. Evaluation of the Surveillance Method

#### 2.2.1. Study Sites and Study Populations

The feasibility of the proposed injury mortality surveillance method was evaluated over one year between February 2019 and January 2020. The evaluation was undertaken in Makwanpur district, south of Kathmandu, with an estimated population of 420,477, with 83% of people living in rural areas. We selected Makwanpur as the study site because it includes three distinct topographical areas (high hills, mid hills and lowland/plains) that are typical of many non-mountainous districts across Nepal. The risk of different types of injuries varies with topography, therefore research conducted here has the potential to be generalisable to many other districts of the country with similar topography [21].

In Nepal, the smallest local government administrative area is known as a municipality. In Makwanpur district, there are eight rural municipalities, one urban municipality and one sub-metropolitan city. We conducted this study in two purposively selected rural municipalities (Bhimphedi and Bakaiya). These municipalities are geographically and socioeconomically representative of other rural municipalities in Makwanpur, but have comparatively poorer access to healthcare facilities and are further away from the main urban centre and the main highway. In these municipalities, there were 31,325 males and 32,677 females living in 12,351 households. There were eight health posts/and two primary healthcare centres, and 108 FCHVs working across these two municipalities [21]. The population of interest included people of any age residing in Bhimphedi and Bakaiya rural municipalities who had died of an injury. Based on the total population of the two rural municipalities [21], we anticipated 39 deaths resulting from an injury over a period of one year (Table 1).

#### 2.2.2. Training

The municipal authorities, 108 FCHVs and 23 staff working in 10 health facilities in the two participating municipality areas were engaged in the study. Two residents of the selected municipalities, who had previously worked on community-based research studies, were recruited as data collectors. Engagement and involvement meetings were held with rural municipality chiefs before starting the field activities. The research team provided training on data collection; one-day training was given to all the FCHVs and focal health facility staff in their respective health post/facility. The FCHVs’ training focused on how to identify deaths at a household level in their area, complete the death notification form and how and when to hand over those forms to their local health facility staff. Health facility staff were trained on how to store the death notification forms securely. Data collectors received two days’ interview and software training at the field office, prior to practicing by entering dummy records onto the software once back in the field.

#### 2.2.3. Piloting and Data Collection

FCHVs gave feedback on the death notification form and the fatal injury inquiry questionnaire was piloted by the data collector with one bereaved family. Following minor modifications, the one year data collection period commenced. All participation in the study was voluntary. The family member was under no obligation to answer the questions for either the death notification form or the fatal injury inquiry questionnaire if they found it upsetting. Should a participant become distressed during the interview, data collection was paused and, when the respondent had recovered, they were offered the choice to either resume or to conclude the interview at that point.

#### 2.2.4. Quality Control and Analysis of Data

A data quality officer at the field office checked for uploaded fatal injury inquiry questionnaire data daily, to ensure that the numerical and free text data had been entered appropriately. A data management officer based remotely at the central research office in Kathmandu provided technical support for REDCap to the data quality officer. There was a monthly meeting with the research team in the UK to review the data collected by that point and to resolve any issues arising. The overall process was evaluated by using the records collected during the data collection period and issues highlighted by FCHVs, health facility staff and data collectors. A monthly feedback meeting with data collectors was also used to inform a review of the method. After completion of the study, analysis of 12 months of data was performed using IBM SPSS statistics Windows V.22.0 (IBM Corp. Armonk, NY, USA) [23]. Frequency calculations and cross-tabulations were used to present descriptive data. Where applicable, the proportion was reported with a 95% confidence interval (95% Confidence Interval).

#### 2.2.5. Evaluation of the Method

To evaluate the feasibility of the method, after completion of data collection and analysis, we held six feedback events in the study area, involving 50 FCHVs and 17 healthcare workers who contributed to the data collection. Feedback focused on ease of use of the tools, the addition of a further task to their workload and whether this impacted on their ability to complete usual activities.

## 3. Results

### 3.1. Feedback from FCHVs and Health Facility Staff

Most FCHVs were able to complete the Death Notification Form without any difficulty. Six out of fifty FCHVs had difficulties with literacy and therefore they sought assistance from family members, other FCHVs or healthcare workers to complete the form when necessary. None of the FCHVs reported difficulties engaging the deceased’s family to complete the form after the mourning period. One of the FCHVs reported finding the Death Notification Form hard at first, but then grew accustomed to it.

The FCHVs and healthcare workers reported that their regular work was not affected by the additional work of collecting death notification data. Rather, it facilitated their routine work because they were able to meet pregnant women and provide them with pregnancy-related information and distribute vitamin A supplements while visiting households. However, some families were cautious when FCHVs asked to see the deceased’s citizenship card to verify their age, fearing this would involve them in legal issues. This was because some families were continuing to receive allowances that the deceased person had been entitled to, and were anxious that providing information would end access to that allowance. Some families were reported to be anxious about disclosing if a death had been suicide for fear that this would lead to police involvement. Healthcare workers reported that some families wondered whether death registration was no longer necessary since the death had been discussed with the FCHV, or whether there was any payment they were entitled to by providing information.

At the end of the evaluation period, FCHVs and health facility workers were pleased to hear the outcomes of the study. They were surprised at the large proportion of deaths that had occurred following an injury and reported being pleased that their contribution had been recognised.

### 3.2. Estimation of Deaths Due to Injury

A total of 457 deaths from all causes were identified from death notification forms over one year (Table 2). Of 457 deaths from all causes, 67 (14.7%) were categorised as secondary to an injury. The proportion of all deaths due to self-harm or assault (intentional injury) were significantly higher (9.6%, 95% CI: 6.9% to 12.3%) than the proportion from unintentional injury (5.0%, 95% CI: 3.0% to 6.0%). Of all-cause deaths, the proportion of injury deaths among males (15.5%, *n* = 41/264) was higher than females (13.5%, *n* = 26/193). Among females, the proportion of self-harm and assault deaths (10.4%, 95% CI: 6.1% to 14.7%) was significantly higher than those due to unintentional injury (3.1%, 95% CI: 0.7% to 5.6%).

**Figure 2:**
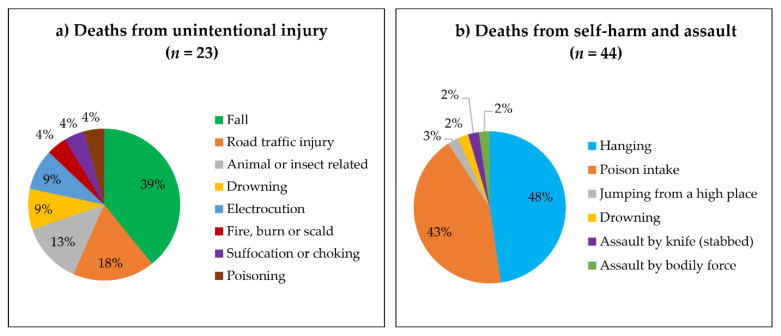
Frequency and proportion of deaths from injuries by injury mechanisms (*n* = 67), (**a**) deaths from unintentional injury, (**b**) deaths from self-harm and assault.

Of the 23 deaths from unintentional injury, 9 (39%) were from falls, followed by road traffic injuries (*n* = 4, 18%) and animal- or insect-related injuries (*n* = 3, 13%). Of 44 deaths from self-harm and assault, 21 (48%) were due to hanging, followed by self-poisoning (*n* = 19, 43%). Of 19 self-poisoning cases, 15 (78.9%) were from agricultural pesticides and four (21.1%) were from insecticides (Figure 2).

Of the 67 injury deaths, 65.7% (*n* = 44) of the injury events occurred at home or in the grounds of the home. Fifty-one (76%) cases died within an hour of being injured. Only 10.4% (*n* = 7) of people received any medical treatment before death. By a median of 57 days (Interquartile Range: 48–72 days) following death, only 25% (*n* = 17) of the cases had their deaths registered in the respective rural municipalities’ ward office.

## 4. Discussion

To our knowledge, this is the first study in Nepal to develop and evaluate a community surveillance method to estimate deaths due to injuries. The method proved feasible to identify and record injury deaths that occurred in a community, employing existing human resources (FCHVs and health post staff) and trained recruited interviewers. In this paper, we presented the methodological detail we used to ensure the completeness of the data collected and the reliability of the methods used to develop the surveillance approach [24].

Other neighbouring nations, such as Bangladesh and India, have also identified the challenges arising from poor-quality fatal injury data [25,26]. In Bangladesh, a household census recorded fatal injuries occurring among household members within the previous year [26], whilst in India, fatal injuries within the previous 6 months were identified through a survey of a representative sample of randomly selected households using verbal autopsy methods [27]. In contrast to these approaches which identified fatal injuries retrospectively over a specified period, our research identified injury deaths prospectively across a geographical area using existing health workers. This is similar to an approach tested in northern India using multipurpose heath workers who, over a period of 6 years, identified over 2300 adult deaths, and from which verbal autopsy methods established the proportion of deaths secondary to injuries as approximately 15% [28]. Prospective approaches using exiting human resources offer a greater potential to establish an ongoing system of estimating injury deaths.

Verbal autopsy (VA) methods developed by the WHO [29] to establish cause of death in settings where access to physicians is limited are now well established. The VA process comprises two phases. First, information is gathered through structured interviews with family members and caregivers of the deceased about their signs, symptoms, medical history and circumstances before death. Second, interview data are interpreted either by physicians (Physician-Certified VA) or, increasingly, by automated methods (Computerised VA Coding), e.g., InterVA and SmartVA, to identify the probable cause(s) of death [13,30]. In contrast, the method described here was designed to also include an earlier step, that is, the identification of deaths in the community. The interview component of our approach is very similar to that used in VA. In the absence of an effective vital registration system and with poor understanding of causes of death, there appears to be a strong argument for the establishment of a community surveillance method in Nepal that could draw on elements of both this study and established VA technology. Whilst our study focused on injury deaths, there is the potential to extend this method to include deaths from all causes.

### 4.1. Key Issues Arising from Our Findings

Potential underreporting of fatal injuries: We expected that 387 deaths and 39 injury deaths would occur in our study area over one year. We identified 457 deaths from all causes, of which 67 (14.6%) deaths were categorised as secondary to an injury. In contrast, the proportion of all deaths that are due to injuries was estimated in a Global Burden of Disease (GBD) study for Nepal to be 9.2% [2]. Our figures suggest that the burden of fatal injuries in Nepal may be underestimated or underreported in published studies, and this warrants further investigation.

High burden of intentional injury deaths: Our results showed that the proportion of deaths from self-harm and assault (intentional injury) was almost double those resulting from unintentional injury (9.6% vs. 5.0% of all-cause deaths). These figures contrast with GBD 2017 estimates for Nepal, which show a higher proportion of deaths due to unintentional injuries (7.7% of all-cause deaths) than intentional injuries (1.6% of all-cause deaths) [2]. The real burden of intentional injuries may have been under reported in the household surveys and census data used by the GBD study to generate estimates for Nepal [11,31]. The stigma and fear of being involved in a legal investigation following a suicide are important factors influencing families’ decisions to report such deaths [32,33].

Limited coverage of death registrations: In Nepal, the Ministry of Federal Affairs and General Administration and the Department of Civil Registration are the official authorities responsible for the death registry. With a federated political system and decentralised structure, ward or municipality offices are the locations of birth, death, marriage, migration and divorce registration units. The Vital Events Registration Act 1976 and Regulation 1977 exert a legal obligation to register a death. People are encouraged to register a death, without charge, up to 35 days after the death [34]. Despite this established system, we found that only 25% (*n* = 17) of this sample of injury fatalities had been registered within a median of 57 days (IQR: 48–72 days) of the death. Factors that may prevent timely and complete coverage of registration of deaths may include the low penalty charges for late registration (less than $1), and that registration is perceived as only necessary when the family require a death certificate (e.g., to legally transfer property or wealth from the deceased). The completeness and coverage of death registrations in Nepal [35] as well as in other South Asian countries is an ongoing issue [34].

### 4.2. Opportunities and Challenges

Throughout the study, full support from the local government, health post staff and local FCHVs facilitated the data collection process. The FCHVs were the backbone of the surveillance system, first because of their community knowledge and existing relationship with families, and second because of their routine regular meetings with health facility staff. Employing local community-based experienced data collectors helped build the trust of the community and simplified the participant recruitment process for interviews. Our experience suggests that the mobilisation of similar existing staff, FCHVs and local data collectors could enable the method to be established in other rural areas across Nepal.

During the evaluation period, we encountered several challenges. Interviewing recently bereaved families requires sensitivity and care, as informants may become upset while recalling the death. We provided training and mock interviews for various types of injury deaths to support data collectors to manage this process professionally. As a result, all informants agreed to be interviewed, and none of the interviews were terminated before completion. Recruiting and training a workforce to conduct interviews with bereaved families would be an important element of establishing and sustaining a community death inquiry system in Nepal.

All FCHVs working in the study area agreed to participate in the project and actively contributed death notification forms during the year. This additional duty was performed alongside their regular duties. Training the FCHVs took 10 days, as researchers travelled to provide training to groups of FCHVs in their own area. Over a year, only two FCHVs left their role. However, both agreed to continue their work until a new FCHV had been assigned to their place. Local health post staff and data collectors provided training to new FCHVs to fill out death notification forms. Most of the FCHVs had no difficulty completing the death notification forms. However, we discovered that a small number of the FCHVs were less literate and had independently sought their family members’ assistance to enable them to participate.

### 4.3. Strengths and Limitations

This study was able to capture all injury deaths that occurred in the study area during the study period. Local government authorities, such as the ward office and health post staff, were consulted and we are not aware that any traumatic cases of death were missed. FCHVs and health facility staff did not find the additional task onerous and could be incorporated into routine practices. The method appears to provide a comprehensive picture of injury deaths. Determining the exact cause of death in our study did not include consultation with a medically trained physician, and therefore, we cannot rule out the misclassification of deaths. The descriptive data on injury deaths identified through this evaluation should not be over interpreted since these results arise from two rural municipalities in one district, so they may not be generalisable to other parts of the country.

### 4.4. Implications for Policy, Practice and Future Research

Death patterns outside the health system are likely to differ from those occurring in health facilities. Lack of accurate, detailed and timely available population-level data on deaths hinders the planning and implementation of health promotion and injury prevention initiatives [36]. Although only evaluated in two wards, we have demonstrated the feasibility of embedding a fatal injury surveillance process within the existing government health system. Further assessment over larger areas appears warranted, as would be the exploration of the potential to include algorithmic assessment of questionnaire responses to determine cause of death, as in Smart VA systems.

Hanging and self-poisoning have been reported as common mechanisms of self-harm in previous Nepalese studies [37,38,39]. Data from these studies were derived from the Hospital Psychiatry Units [39], medical records [38] or following autopsies in Police investigation cases [37]. Our findings suggest that fatal intentional harm is common in rural communities and may not involve healthcare systems. We suggest that further studies are needed to identify the factors leading to self-harm, in particular those resulting in poisoning and hanging.

Collaborative and multisectoral approaches at local and national levels are essential to improve the coverage and functionality of the death registration system [40]. Our study revealed that many injury deaths in rural communities are likely to be unregistered, and therefore unrecognised by health systems due to the absence of a functioning civil registration system [41]. In such situations, it is challenging to determine the cause of death with some degree of accuracy and confidence [8]. We present an argument for investment in a robust and complete vital registration system in Nepal.

## 5. Conclusions

This study has shown that conducting fatal injury surveillance in rural Nepal is feasible and has the potential to yield valuable data. In the absence of a robust national death registration system, an alternative form of injury-related death identification is warranted. The method developed and evaluated in our study may inform future methods for identifying, assessing and monitoring trends in injury deaths in rural Nepal and other LMICs.

## Figures and Tables

**Table 1:** Estimated number of all-cause deaths and injury deaths in the study area over a period of one year.

Two Rural Municipalities	Total Population	Total Deaths (Expected)
Total population	64,602	
Any deaths estimated	0.6% * of 64,602	387
Injury death expected	10% ** of 387	39

* Nepal crude death rate: 6 per 1000 population [22]. ** Based on WHO estimates that globally ~9% of deaths are secondary to injuries [1].

**Table 2:** Frequency and proportion of deaths from injuries of all-cause deaths over a one-year period.

All-Cause Deaths	Deaths from Unintentional Injury *		Deaths from Self-Harm and Assault **		All-Injury Deaths	
	*n*	Proportion (95% CI)	*n*	Proportion (95% CI)	*n*	Proportion (95% CI)
Overall (*n* = 457)	23	5.0 (3.0–6.0)	44	9.6 (6.9–12.3)	67	14.6 (11.4–17.9)
Age (years), median (IQR)	-	55 (19–65)	-	36.5 (25.3–53.5)	-	40 (24–56)
Male (*n* = 264)	17	6.4 (3.5–9.4)	24 ^†^	9.1 (5.6–12.6)	41	15.5 (11.2–19.9)
Female (*n* = 193)	6	3.1 (0.7–5.6)	20 ^‡^	10.4 (6.1–14.7)	26	13.5 (8.7–18.3)

IQR = Interquartile Range, CI = Confidence Interval, * ‘Unintentional injury’ includes road traffic injury, falls, burns, poisoning, drowning, animal injuries, cuts and lacerations, suffocation, choking or electrocution. ** ‘Self-harm and assault’ includes any physical trauma inflicted on oneself or another; ^†^ of 24 males, 14 committed self-harm by hanging and seven by self-poisoning and three by other mechanisms; ^‡^ of 20 females, 12 committed self-harm by self-poisoning, seven by hanging and one by other mechanism.

## Data Availability

The datasets used and/or analysed during the current study are available from the corresponding author on reasonable request.

## Supplementary Materials

The following are available online at https://www.mdpi.com/article/10.3390/ijerph18178912/s1, Table S1: Death Notification Form, Table S2: Fatal Injury Inquiry Questionnaire.
