# Supplementary material for: Development and Evaluation of a Community Surveillance Method for Estimating Deaths Due to Injuries in Rural Nepal
Source: Int J Environ Res Public Health. 2021 Aug 24;18(17):8912. doi: 10.3390/ijerph18178912 (PMC8430737; doi:10.3390/ijerph18178912)
Supplement: Supplementary file 1 [file ijerph-18-08912-s001.zip › ijerph-1320402-supplementary.pdf]

Supplementary Materials

Table S1: Death Notification Form

Death Notification Form

**Deceased's address in Makwanpur district**					
**Name of rural municipilaty:**					
**Ward number: ____ ____**					
**Village/Tole:**					
**Case ID number: RMN [ ] WN [ ] IN [ ] (**To enter de-identifiable data into a Fatal Injury Inquiry Questionnaire, this ID will be created and used)					
For the Female Community Health Volunteer : Read the consent statement below to the respondent. Ask the respondent if he or she has any questions. Once any questions are answered, ask the respondent if he or she is willing to take part in the study.<br><br>I am helping a research team from the Nepal Injury Research Centre (NIRC). I have been informed that [name of deceased] has recently died. I am very sorry to hear that [a member of your household/your friend/your colleague] has passed away recently. Please accept my sympathies. For the purpose of better understanding and improving health, we are collecting information on all recent deaths in this area. If it is OK with you and your family at this difficult time, I would like to ask some questions to the main caretaker of [the deceased's name] about them and what happened before they died. I will pass the information you give me to the research team, who may wish to contact you for more information at a later time. Are you willing to answer some questions about [name of deceased]?					
**[What is the name of respondent?]**					
First Name:		Surname:		Contact number:	
**[Date of consent sought from respondent]**					
		Day: ___ ___ Month: ___ ___ Year: ___ ___			
**What was the first and surname (or family name) or given name(s) of the deceased?**					
First Name:		Surname (or family name):			
**[Full name and ID number of the FCHV]**					
Name:		ID number: ___ ___			
**[Did respondent give consent?]**					
If answer is "Yes" proceed to fill out the rest of the form.				☐	Yes
If answer is "No" then thank respondent for their time and leave.				☐	No
**General Information on the deceased and screening questions**					
**What was the gender of deceased?**					
☐	Female	☐	Male	☐	Refuse to answer
**Is the date of birth (DoB) known?**					
☐	Yes	☐	No	☐	Refuse to answer
**When was the deceased born?**					
Day: ___ ___ Month: ___ ___ Year: ___ ___					
**Is the date of death known?**					
☐	Yes	☐	No	☐	Refuse to answer
**When did (s) he died?**					
Day: ___ ___ Month: ___ ___ Year: ___ ___					
**What was the last known age of the deceased?**					
☐	Years (if ≥1 year): ___ ___	☐	Months (if <1 year): ___ ___	☐	Days (if <1 month): ___ ___
**Can you please tell me about what happened to [name of deceased] when (s) he died? [Free text below]**					
**[What was the cause of death]** (this will be decided according to the above free text informtion)					
☐	Injury or violence	☐	Communicable or infecticious disease	☐	Non-comminicable disease
☐	Unknown	☐	Others (specify:)		
**[Give thanks to respondent for their time and providing information] [Pass this form to the Health Post in Charge at your next monthly meeting]**					

Table S2: Fatal Injury Inquiry Questionnaire

**Fatal injury inquiry Questionnaire**				
INTERVIEWER: Read the consent form to the respondent. Ask the respondent if he or she has any questions. Once any questions are answered, ask the respondent if he or she is willing to take part in the study.<br><br>My name is [your name]. I am an interviewer with the Nepal Injury Research Centre (NIRC) project. I have been informed that [name of deceased] has recently died. I am very sorry to hear that [a member of your household/family, your friend, your colleague] has passed away recently. Please accept my sympathies. For the purpose of understanding and improving health , we are collecting information on all recent injury deaths in this area. If it is OK with you and your family at this difficult time, I would like to talk to the main caretaker of [the deceased's name] and ask some questions about the events that happened before [the deceased's name] died.				
**[Did respondent give consent?]**		Code		
		1	Yes	
		2	No	
If answer is "Yes" proceed interview.<br>*If answer is "No" then thank respondent for their time and end the interview*				
**Case ID number: RMN [ ] WN [ ] IN [ ]** (RMN = Rural Municipality Name; WN = Ward Number; IN = Interview Number)<br>**(**To enter non-identifiable data into a VA questionnaire, this ID will be created and used)<br>10 01 01 for Bakaiya and 20 01 01 for Bhimphedi (starting case ID numbers)				
**[Date of interview]**				
		Day: ___ ___ Month: ___ ___ Year: ___ ___ (Nepali dates)		
**[Interview start time]**				
Record time at start of interview (24 hours clock)		Hours: ___ ___ Minutes: ___ ___		
**[Full name and ID number of VA interviewer]**				
		Name: ______________________________		
		ID number: ___ ___ (e.g. BAK 01 and BHI 02)		
**ID**	**Question**		**Answer (s)**	**Skip To**
Questions to be read to the respondent are in **bold**				
[Questions that are NOT to be read to respondent are in brackets]				
Hints to the interviewer are in non-bold and *italicized* text below relevant questions				
The embedded skip patterns will ensure that only the relevant subset of questions is applied				
**Section 1: Information on the respondent**				
**101**	**What is your/the respondent's relationship to the deceased?**		Please circle the appropriate number/s	
	First verify if the respondent is a family member, and only if it is not a family member choose the other categories like health worker or public official.			
		1	Parent	102
		2	Child	102
		3	Other family member (specify ____)	102
		4	Friend	102
		5	Health worker	102
		6	Public official	102
		97	Other ____________________	102
		99	Refused to answer	102
**102**	**[What is the respondent's gender]**			
		1	Female	103
		2	Male	103
**103**	**What is your/the respondent's age**			
			In Years: ___ ___	104
**104**	**Did you/the respondent live with the deceased in the period leading to her/his death?**			

		1	Yes	Next Q.
		2	No	Next Q.
		98	Doesn't know	Next Q.
		99	Refused to answer	Next Q.

Can you please tell me about what happened to [name of deceased] when (s) he died?
A breif description of injury death by respondent will allow the interviewer time to think about which sections of the form they are going to need to fill in [**Free text]**

____

Section 2: Information on the Deceased

**201**	**Gender of deceased**			
		1	Female	202
		2	Male	202
**202**	**What was the last known age of the deceased?**			
		☐	Years (if ≥1 year): ___ ___	203
		☐	Months (if <1 year): ___ ___	203
	(Record "00" days if less than one day)	☐	Days (if <1 month): ___ ___	203
**203**	**What was the deceased's ethnic group?**			
		1	Badhe	204
		2	Badi	204
		3	Bahae	204
		4	Banda	204
		5	Bangali	204
		6	Baniya	204
		7	Bantar	204
		8	Baramu	204
		9	Bhediyar	204
		10	Bhote	204
		11	Bing	204
		12	Bote	204
		13	Brahman (Hill)	204
		14	Brahman (Terai)	204
		15	Byansi	204
		16	Chamar	204
		17	Chepang	204
		18	Chhantal	204
		19	Chhetri	204
		20	Chidimar	204
		21	Churaute	204
		22	Damai	204
		23	Danuwar	204
		24	Darai	204
		25	Dhangad/Jhagad	204
		26	Dhanuk	204
		27	Dhimal	204

		28	Dhobi	204
		29	Dhunia	204
		30	Dhusadh/Paswan	204
		31	Dom	204
		32	Dura	204
		33	Gangai	204
		34	Ganine	204
		35	Gharti/Bhujel	204
		36	Gurung	204
		37	Hajaam	204
		38	Halkhor	204
		39	Haluwai	204
		40	Hayu	204
		41	Hyolomu	204
		42	Jain	204
		43	Jirel	204
		44	Kahar	204
		45	Kalwar	204
		46	Kamarm	204
		47	Kami	204
		48	Kanu	204
		49	Kayastha	204
		50	Kewat	204
		51	Khatway	204
		52	Kisan	204
		53	Koche	204
		54	Koiri	204
		55	Kumal	204
		56	Kumhar	204
		57	Kurmi	204
		58	Kusunda	204
		59	Lepcha	204
		60	Limbu	204
		61	Lodhar	204
		62	Lohar	204
		63	Magar	204
		64	Majhi	204
		65	Mali	204
		66	Mallah	204
		67	Marwadi	204
		68	Meche	204
		69	Munda	204
		70	Mushar	204

		71	Muslim	204
		72	Newar	204
		73	Nuniya	204
		74	Nurang	204
		75	Pahari	204
		76	Patharkatta/Kusbadiya	204
		77	Rai	204
		78	Rajbansi	204
		77	Rajbar	204
		80	Raji	204
		81	Rajput	204
		82	Raute	204
		83	Santhal/Satar	204
		84	Sanyasi	204
		85	Sarki	204
		86	Sherpa	204
		87	Sonar	204
		88	Sudhi	204
		89	Sunwar	204
		90	Tajpuriya	204
		91	Tamang	204
		92	Tatma	204
		93	Teli	204
		94	Thakali	204
		95	Thakur	204
		96	Thakuri	204
		97	Thami	204
		98	Tharu	204
		99	Walung	204
		100	Yadav	204
		101	Yakkah	204
		9997	Other ___________________	204
		9998	Doesn't know	204
		9999	Refused to answer	204
**204**	**Where did the deceased die?**			
		1	Hospital	205
		2	Other heath facility	205
		3	Home	205
		4	On route to hospital or facility	205
		97	Other ___________________	205
		98	Doesn't know	205
		99	Refused to answer	205
**205**	**Do you have a death registration certificate?**			
		1	Yes	206

		2	No	Section 3.1
**206**	**[Death registration number/certificate]**			
	Enter a "NA" if this information is not available			
	___________________________________			207
**207**	**[Is the date of registration available?]**			
		1	Yes	208
		2	No	209
		98	Doesn't know	209
**208**	**[Date of registration]**			
	Day: ___ ___ Month: ___ ___ Year: ___ ___			209
**209**	**[Place of registration]**			
	Enter a "NA" if this information is not available		______________________________	Section 3.1
	**Section 3.1: Socoio-economic information** (Skip to section 3.2 if deceased was **LESS** than 12 years old)			
**301.1**	**What was the level of education of the deceased?**			
		1	No formal education	302.1
		2	Primary school	302.1
		3	Secondary school	302.1
		4	Post-secondary	302.1
		98	Doesn't know	302.1
		99	Refused to answer	302.1
**302.1**	**What was marital status of deceased?**			
		1	Never married	303.1
		2	Married	303.1
		3	Separated	303.1
		4	Divorced	303.1
		5	Widowed/widower	303.1
		98	Doesn't know	303.1
		99	Refused to answer	303.1
**303.1**	**What was her/his economic activity status in year prior to death?**			
	The deceased might have had several activities. Choose the one that was probabaly true for most of the year preceding illness and death			
		1	Mainly unemployed	305.1
		2	Mainly employed (salary job)	304.1
		3	Skilled worker/waged labourer	305.1
		4	Agriculture	305.1
		5	Business	305.1
		6	Pensioner	305.1
		7	Student	305.1
		97	Other ___________________	305.1
		98	Doesn't know	305.1
		99	Refused to answer	305.1
**304.1**	**If mainly employed, what was her/his occupation, that is, what kind of work did (s)he mainly do?**			
	____			305.1
**305.1**	**What is the monthly income of your household?**			
	Overall household income in average per month	☐	Roughly NRs. _______________	Section 3.2

		1	Doesn't know	Section 3.2
		2	Refused to answer	Section 3.2
**Section 3.2: Socoio-economic information** (Skip to section 4 if deceased was **MORE** than 12 years old)				
	If father present	☐	Applicable	301.2
	If father not present	☐	Not applicable	304.2
**301.2**	**What was the highest level of education of the father?**			
		1	No formal education	302.2
		2	Primary school	302.2
		3	Secondary school	302.2
		4	Post-secondary	302.2
		98	Doesn't know	302.2
		99	Refused to answer	302.2
**302.2**	**What was the father's economic activity status in year prior to his child's death?**			
		1	Mainly unemployed	304.2
		2	Mainly employed (salary job)	303.2
		3	Skilled worker/waged labourer	304.2
		4	Agriculture	304.2
		5	Business	304.2
		6	Pensioner	304.2
		7	Student	304.2
		97	Other ___________________	304.2
		98	Doesn't know	304.2
		99	Refused to answer	304.2
**303.2**	**What was the father's occupation, that is, what kind of work did he mainly do?**			
	____			
	If mother present	☐	Applicable	304.2
	If mother not present	☐	Not applicable	Section 4
**304.2**	**What was the highest level of education of the mother?**			
		1	No formal education	305.2
		2	Primary school	305.2
		3	Secondary school	305.2
		4	Post-secondary	305.2
		98	Doesn't know	305.2
		99	Refused to answer	305.2
**305.2**	**What was the mother's economic activity status in year prior to her child's death?**			
		1	Mainly unemployed	307.2
		2	Mainly employed (salary job)	306.2
		3	Skilled worker/waged labourer	307.2
		4	Agriculture	307.2
		5	Business	307.2
		6	Pensioner	307.2
		7	Student	307.2
		97	Other ___________________	307.2
		98	Doesn't know	307.2

		99	Refused to answer	307.2
**306.2**	**What was the mother's occupation, that is, what kind of work did she mainly do?**			
	____			307.2
**307.2**	**What is the monthly income of your household?**			
	Overall household income in average per month	☐	Roughly NRs. ________________	Section 4
		98	Doesn't know	Section 4
		99	Refused to answer	Section 4
**Section 4: History of injuries/accidents**				
**401**	**What was the intent of injury that led to her/his death?**			
	[This question is to identify intent of injury]			
		1	Unintentional (accidental)	402
		2	Intentional (self-harm/sucide)	403
		3	Intentional (assault/violence)	404
		97	Other ___________________	Section 5
		98	Doesn't know	Section 5
		99	Refused to answer	Section 5
**402**	**If it was unintentional, what was the mechanism?**			
		1	Road traffic injury	405 a - d
		2	Fall	406 a - d
		3	Poisoning	407 a - b
		4	Drowning and submersion	408 a - b
		5	Animal/insect bite/sting	409 a - b
		6	Fire, burn or scald	410 a - f
		7	Electrocution	411 a
		8	Stabbed, cut or pierced	412 a
		9	Blunt object	413 a - b
		10	Suffocation/choking/asphyxia	414 a
		97	Other ____________________	Section 5
		98	Doesn't know	Section 5
		99	Refuse to answer	Section 5
**403**	**If it was intentional injury (self-harm/sucide), what was the mechanism?**			
	Includes purposely self-inflicted poisoning or injury, suicide (attempted)			
		1	Crashing of motor vehicle	Section 5
		2	Jumping from a high place	Section 5
		3	Jump in front of moving object	Section 5
		4	Poisoning	407 a - b
		5	Drowning and submersion	408 a - b
		6	Fire, burn or scald	410 a - f
		7	Electrocution	411 a
		8	Stabbed, cut or pierced	412 a
		9	Blunt object	413 a – b
		10	Hanging, strangulation, suffocation	414_a
		11	Firearm or gun	Section 5
		97	Other ____________________	Section 5

		98	Doesn't know	Section 5
		99	Refuse to answer	Section 5
**404**	**If it was intentional injury (assault/violence), what was the mechanism?**			
	Includes homicide injuries inflicted by another person with intent to injure or kill, by any means			
		1	Crashing of motor vehicle	Section 5
		2	Pushing from a high place	Section 5
		3	Pushing in front of moving object	Section 5
		4	Poisoning	407 a - b
		5	Drowning and submersion	408 a - b
		6	Fire, burn or scald	410 a - f
		7	Electrocution	411 a
		8	Stabbed, cut or pierced	412 a
		9	Blunt object	413 a – b
		10	Hanging, strangulation, suffocation	414_a
		11	Firearm or gun	Section 5
		12	Bodily force (physical violence, fight)	Section 5
		13	Sexual assault by bodily force (rape)	Section 5
		97	Other ____________________	Section 5
		98	Doesn't know	Section 5
		99	Refused to answer	Section 5
**405_a**	**Mode of Transport**			
		1	Pedestrian	405 b
		2	Non-engine vehicle (e.g.bicycle)	405 b
		3	Motorcycle, Scooter, Moped	405 b
		4	Auto-ricky, Tempo, Magic	405 b
		5	Car, Jeep, Microbus	405 b
		6	Bus, Mini-bus	405 b
		7	Truck, Lorry, Tipper	405 b
		97	Other ____________________	405 b
		98	Doesn't know	405 b
		99	Refused to answer	405 b
**405_b**	**Type of road user: What was the role of injured person who died later?**			
		1	Pedestrian	405 c
		2	Driver (or operator)	405 c
		3	Passenger	405 c
		97	Others ____________________	405 c
		98	Doesn't know	405 c
		99	Refused to answer	405 c
**405_c**	**Was there a collision?**			
		1	Yes	405 d
		2	No	Section 5
		98	Doesn't know	Section 5
		99	Refused to answer	Section 5
**405_d**	**In the RTA, what was the person in collision with?**			
		1	Non-engine vehicle (e.g.bicycle)	Section 5
		2	Motorcycle, Scooter, Moped	Section 5

		3	Auto-ricky, Tempo, Magic	Section 5
		4	Car, Jeep, Microbus	Section 5
		5	Bus, Mini-bus	Section 5
		6	Truck, Lorry, Tipper	Section 5
		97	Other ___________________	Section 5
		98	Doesn't know	Section 5
		99	Refused to answer	Section 5
**406_a**	**What was the type of fall?**			
	This includes accidents and cases where it is unknown if it was an accident or whether there was intentional violence.			
		1	Same level	406 b
		2	Different level	406 c
		98	Doesn’t know	Section 5
		99	Refused to answer	Section 5
**406_b**	**For the fall on same level, where was the location?**			
		1	Bathroom	407 a
		2	Bedroom/livingroom	407 a
		3	Kitchen	407 a
		4	Street/road	407 a
		5	School	407 a
		6	Workplace	407 a
		7	Play ground	407 a
		97	Other ____________________	407 a
		98	Doesn’t know	407 a
		99	Refuse to answer	407 a
**406_c**	**For the different level fall, where was the location?**			
		1	Stairs or ladder	
		2	Tree	406 d
		3	Roof of house	406 d
		4	Furniture	406 d
		5	Back of animal	406 d
		6	From the vehicle	406 d
		7	High terrain/hill	406 d
		97	Other ____________________	406 d
		98	Doesn’t know	406 d
		99	Refuse to answer	406 d
**406_d**	**From what height was the fall?**			
		1	<1 metre	Section 5
		2	1-5 metre	Section 5
		3	6-10 metre	Section 5
		4	>10 metre	Section 5
		97	Other ____________________	Section 5
		98	Doesn’t know	Section 5
		99	Refuse to answer	Section 5
**407_a**	**What was the category of the poisoning substance?**			

	This includes accidents and cases where it is unknown if it was an accident or whether there was intentional violence.			
		1	Agricultural Pesticide<br>If known, Name: __________________<br>Doesn't know Name	407 b
			Agricultural additive or fertilizer<br>If known, Name: __________________<br>Doesn't know Name	407 b
		2	Other Insecticide<br>If known, Name: __________________<br>Doesn't know Name	407 b
		3	Rodenticides<br>If known, Name: __________________<br>Doesn't know Name	407 b
		4	Sleeping pills	407 b
		5	Soap/detergent	407 b
		6	kerosene	407 b
		7	Antiseptic	407 b
		8	Other medicines	407 b
		9	Wild plant<br>If known, Name: __________________<br>Doesn't know Name	407 b
		97	Other ______________________	407 b
		98	Doesn't know	407 b
		99	Refuse to answer	407 b
**407_b**	**What was the nature of the container?**			
		1	Original container	Section 5
		2	Not in original container	Section 5
		98	Doesn't know	Section 5
		99	Refused to answer	Section 5
**408_a**	**What was the water source where the drowning occured?**			
	This includes accidents and cases where it is unknown if it was an accident or whether there was intentional violence.			
		1	Pond or Lake	408 b
		2	Ditch	408 b
		3	Well	408 b
		4	River/stream	408 b
		5	Canal	408 b
		6	Uncovered water container	408 b
		97	Other ______________________	408 b
		98	Doesn't know	408 b
		99	Refuse to answer	408 b
**408_b**	**Did the person know how to swim?**			
		1	Yes	Section 5
		2	No	Section 5
		98	Doesn't know	Section 5
		99	Refused to answer	Section 5
**409_a**	**What was the animal/insect?**			

	This includes accidents and cases where it is unknown if it was an accident or whether there was intentional violence.			
		1	Dog	409 b
		2	Cat	409 b
		3	Snake	409 b
		4	Scorpion	409 b
		5	Hornet/wasp/bees	409 b
		6	Cattle/buffalo	409 b
		7	Wild animal	409 b
		97	Other ____________________	409 b
		98	Doesn't know	409 b
		99	Refuse to answer	409 b
**409_b**	**How did these animals/insects cause the injury?**			
		1	Bite	Section 5
		2	Sting	Section 5
		3	Kick	Section 5
		4	Crushed/stepped on	Section 5
		5	Knocked/impaled/butted with horn	Section 5
		97	Other ____________________	Section 5
		98	Doesn't know	Section 5
		99	Refuse to answer	Section 5
**410_a**	**What was the cause of the burn/scald?**			
		1	Flame	410 b
		2	Hot liquid or steam	410 c
		3	Hot object	410 d
		4	Explosive	410 e
		5	Chemical	410 f
		97	Other ____________________	Section 5
		98	Doesn't know	Section 5
		99	Refuse to answer	Section 5
**410_b**	**What was the source of flames?**			
		1	Cooking fire	Section 5
		2	Heating fire	Section 5
		3	Workplace sources	Section 5
		4	Kerosene/oil lamp	Section 5
		5	Matches/ gas lighter	Section 5
		6	Candle lamp/lights	Section 5
		7	Electric short circuit	Section 5
		97	Other ____________________	Section 5
		98	Doesn't know	Section 5
		99	Refuse to answer	Section 5
**410_c**	**What was the hot liquid or steam?**			
		1	Cooking water/steam	Section 5
		2	Bathing water	Section 5
		3	Cooking oil	Section 5
		4	Tea/coffee/milk	Section 5
		5	Daal/soup/liquid food	Section 5
		6	Workplace hot water/steam source	Section 5

		97	Other _____________________	Section 5
		98	Doesn't know	Section 5
		99	Refuse to answer	Section 5
**410_d**	**What was the hot object?**			
		1	Cooking utensils/pan	Section 5
		2	Coal/ fuel	Section 5
		3	Iron/metal	Section 5
		4	Muffler/ engine part	Section 5
		5	Heater/radiator	Section 5
		6	Oven/kiln	Section 5
		7	Workplace source	Section 5
		8	Hot ashes	Section 5
		9	Iron [clothes]	Section 5
		97	Other _____________________	Section 5
		98	Doesn't know	Section 5
		99	Refuse to answer	Section 5
**410_e**	**Why was the explosive used?**			
		1	Fireworks/crackers	Section 5
		2	Construction works	Section 5
		3	Pressure cooker	Section 5
		4	LPG cylinder	Section 5
		5	Paraffin stove	Section 5
		6	Violence/riots	Section 5
		7	Abandoned/IED	Section 5
		97	Other _____________________	Section 5
		98	Doesn't know	Section 5
		99	Refuse to answer	Section 5
**410_f**	**What was the chemical that caused the burn?**			
		1	Acid	Section 5
		2	Lime	Section 5
		3	Antiseptic	Section 5
		97	Other _____________________	Section 5
		98	Doesn't know	Section 5
		99	Refuse to answer	Section 5
**411_a**	**What was the source of the electric shock?**			
		1	Electrical use in the home	Section 5
		2	Electrical use outside home	Section 5
		3	Lightning	Section 5
		4	Generator/Solar	Section 5
		97	Other _____________________	Section 5
		98	Doesn't know	Section 5
		99	Refuse to answer	Section 5
**412_a**	**Stabbed, cut or pierced: what was sharp object?**			
		1	Knife	Section 5
		2	Sickle	Section 5
		3	Scissors	Section 5
		4	Axe	Section 5
		5	Spade	Section 5
		6	Broken glass	Section 5

		7	Hoe/spikes	Section 5
		8	Other household object	Section 5
		9	Bamboo/wooden articles	Section 5
		10	Bladed wire/fence	Section 5
		97	Other ____________________	Section 5
		98	Doesn't know	Section 5
		99	Refuse to answer	Section 5
**413_a**	**What was the blunt object?**			
		1	A Moving object	413 b
			If known, specify ____________________ *If not known, enter "98"*	413 b
		2	A fixed object	413 b
			If known, specify ____________________ *If not known, enter "98"*	413 b
		97	Other ____________________	413 b
		98	Doesn't know	413 b
		99	Refuse to answer	413 b
**413_b**	**What was that blunt object used for?**			
		1	Farm work	Section 5
		2	Household works	Section 5
		3	Factory works	Section 5
		4	At the shop	Section 5
		5	Construction work	Section 5
		6	Office works	Section 5
		7	Sports/game	Section 5
		97	Other ____________________	Section 5
		98	Doesn't know	Section 5
		99	Refuse to answer	Section 5
**414_a**	**What was the suffocating or choking agent?**			
		1	Hanging	Section 5
		2	Smoke/carbon monoxide	Section 5
		3	Other gas ________________	Section 5
		4	Liquid ______________	Section 5
		5	Covered by clothes	Section 5
		6	Covered by adult body	Section 5
		7	Covered by earth	Section 5
		8	Covered by plastic	Section 5
		9	Food items	Section 5
		10	Small objects (e.g. Coins)	Section 5
		97	Other ____________________	Section 5
		98	Doesn't know	Section 5
		99	Refuse to answer	Section 5
**Section 5: Injury detail regardless of injury type**				
**501**	**Where was (s)he when (s)he sustained the injury?**			
		1	Home / compound	502
		2	School	502
		3	Work place	502

		4	Highway/road/street	502
		5	Recreational area	502
		97	Other ____________________	502
		98	Doesn't know	502
		99	Refuse to answer	502
**502**	**What was (s)he doing at the time (s)he sustained the injury?**			
		1	Work	503
		2	Education	503
		3	Leisure / play	503
		4	Organised Sports	503
		5	Travelling to or from school or work	503
		6	Travelling for other purpose	503
		97	Other ____________________	503
		98	Doesn't know	503
		99	Refuse to answer	503
**503**	**Do you know when (s)he sustained the injury?**			
		1	Yes	504
		2	No	Section 6
		98	Doesn't know	Section 6
		99	Refused to answer	Section 6
**504**	**When did (s)he suatained injury?**			
	Hours: ___ ___ (24 hours clock) Day: ___ ___ Month: ___ ___ Year: ___ ___			505
**505**	**How long was the time between the injury being sustained and when the death occurred?**			
	This can be cross-cheked with the date of injury sustained and date of death.	1	Died within an hour	Section 6
		2	Hours: ___ ___	Section 6
		3	Days : ___ ___	Section 6
		4	Months: ___ ___	Section 6
		5	Years: ___ ___	Section 6
		98	Doesn't know	Section 6
		99	Refused to answer	Section 6
**Section 6: Health service utilization and cost associated with injury**				
**601**	**Did (s)he receive any treatment for the injury?**			
		1	Yes	603
		2	No	602
		98	Doesn't know	Section 7
		99	Refused to answer	Section 7
**602**	**Why was no treatment taken?**			
	Check all that apply			
		1	Person had already died	Section 7
		2	Did not think treatment was necessary	Section 7
		3	Did not know where to go	Section 7
		4	Did not go because it was too far	Section 7
		5	Vehicle not available	Section 7
		6	No one available to accompany at the time	Section 7
		7	Insufficient time	Section 7
		8	Lack of available money	Section 7

		9	Barrier from the family	Section 7
		10	Was not possible to travel due to night/natural disaster (any time)	Section 7
		11	Quality of care was not good at the health care centre	Section 7
		12	No one was available at the health care centre	Section 7
		97	Other ____________________	Section 7
		98	Doesn't know	Section 7
		99	Refuse to answer	Section 7
**603**	**Was care sought outside the home when (s)he had the injury?**			
		1	Yes	604
		2	No	Section 7
		98	Doesn't know	Section 7
		99	Refused to answer	Section 7
**604**	**Where or from whom did you seek care?**			
	Check all that apply			
		1	Traditional healer	609
		2	Homeopath	609
		3	Religious leader	609
		4	Government hospital	605
		5	Health-posts or PHCC	605
		6	Private hospital	605
		7	Private physician's clinic	605
		8	Relative, friend (outside household)	609
		9	Pharmacy	609
		97	Other ____________________	609
		98	Doesn't know	609
		99	Refuse to answer	609
**605**	**What was the name and address of any hospital, health center or clinic where the care was sought?**			
	1st visited - Name: ____________________Address:____________________________________			606
	2nd visited - Name:____________________Address:____________________________________			606
	3rd visited - Name:____________________Address:____________________________________			606
**606**	**Did (s)he use any transport to get to the health facility?**			
		1	Yes	607
		2	No	610
		98	Doesn't know	610
		99	Refused to answer	610
**607**	**What means of transportation was used to travel to the health facility?**			
		1	Ambulance	608
		2	Bus/truck/tractor	608
		3	Jeep, Car or Taxi	608
		4	Motorbike	608
		5	Non motorised vehicle (e.g. Bicycle)	608
		6	Proper stretcher	608

		7	Locally made stretcher	608
		8	Carried by person	608
		97	Other ____________________	608
		98	Doesn't know	608
		99	Refuse to answer	608
**608**	**How long did it take to get to the first health facility attended by the deceased person?**			
		☐	(If an hour or more) Hours: ___<br>(If less than an hour) Minutes: ___	609
		98	Doesn't know	610
		99	Refused to answer	610
**609**	**What was the associated costs for all transportation to seek care (excluding medical expenses)?**			
		☐	Roughly NRs. __________	610
		☐	Doesnt know	610
**610**	**What was the overall hospitalization and or treatment and medical care costs or other costs of caring for them (excluding transportation)?**			
			Roughly NRs. _________	611
			Doesn't know	611
**611**	**Over the course of injury, did the total costs of care and treatment prohibit other household payments?**			
		1	Yes	612
		2	No	Section 7
		98	Doesn't know	Section 7
		99	Refused to answer	Section 7
**612**	**Did a family member have to borrow money to pay for medical treatment for the injury or to make up for loss of income of the injured person?**			
		1	Yes	613
		2	No	Section 7
		98	Doesn't know	Section 7
		99	Refused to answer	Section 7
**613**	**If money was borrowed, how much?**			
			Roughly NRs. _______________	Section 7

Section 7: Open narrative

**701**	**Thank you very much for your information and time. I would like to end this interview here. If you have any things to say that you think we have missed, please feel free say it now.**	
	Record detailed notes of response; use additional paper as needed. If needed, probe for additional details on when respondent recognized symptoms, care sought, barriers to care, issues with transport, abnormalities, etc.	End

____

____

**[Interview end time]**	
Record time at end of interview (24 hours clock)	Hours: ___ ___ Minutes: ___ ___

Report End
